# Probiotic supplementation for 24 weeks in patients with non-alcoholic steatohepatitis: the PROBILIVER randomized clinical trial

**DOI:** 10.3389/fnut.2024.1362694

**Published:** 2024-03-27

**Authors:** Amanda Souza Silva-Sperb, Helena Abadie Moraes, Samantha Thifani Alrutz Barcelos, Bruna Concheski de Moura, Larisse Longo, Matheus Truccolo Michalczuk, Carlos Thadeu Schmidt Cerski, Carolina Uribe-Cruz, Themis Reverbel da Silveira, Mário Reis Álvares-da-Silva, Valesca Dall’Alba

**Affiliations:** ^1^Graduate Program in Gastroenterology and Hepatology, Universidade Federal do Rio Grande do Sul, Porto Alegre, Brazil; ^2^Experimental Laboratory of Hepatology and Gastroenterology, Experimental Research Center, Hospital de Clínicas de Porto Alegre, Porto Alegre, Brazil; ^3^Division of Gastroenterology, Hospital de Clínicas de Porto Alegre, Porto Alegre, Brazil; ^4^Unit of Surgical Pathology, Hospital de Clínicas de Porto Alegre, Porto Alegre, Brazil; ^5^Nutrition Division, Hospital de Clínicas de Porto Alegre, Porto Alegre, Brazil

**Keywords:** non-alcoholic fatty liver disease, probiotics, toll-like receptor-4, cytokeratin 18, metabolic syndrome, microbiota, metabolic dysfunction-associated steatotic liver disease

## Abstract

**Background and aim:**

Considering the increasing prevalence of non-alcoholic steatohepatitis (NASH) and treatment gaps, this study aimed to evaluate the effect of probiotic supplementation on liver function markers, nutritional status, and clinical parameters.

**Methods:**

This double-blind, randomized clinical trial (ClinicalTrials.gov ID: NCT0346782) included adult outpatients with biopsy-proven NASH. The intervention consisted of 24 weeks of supplementation with the probiotic mix *Lactobacillus acidophilus* (1 × 10^9^ CFU) + *Lactobacillus rhamnosus* (1 × 10^9^ CFU) + *Lactobacillus paracasei* (1 × 10^9^ CFU) + *Bifidobacterium lactis* (1 × 10^9^ CFU), or placebo, twice a day. The following parameters were evaluated: demographic and clinical data, transient elastography (FibroScan), liver enzymes, NAFLD *fibrosis score*, fatty liver index, laboratory assessment, serum concentration of toll-like receptor-4 (sTLR-4) and cytokeratin 18 (CK-18), anthropometric data, dietary intake, and physical activity. Regarding data analysis, the comparison between the groups was based on the delta of the difference of each variable analyzed (value at the end of treatment minus the baseline value) using the *t*-test for independent samples or the Mann–Whitney *U*-test.

**Results:**

Forty-four patients with NASH completed the trial (51.4 ± 11.6 years). At baseline, 87% of participants had a mild liver fibrosis degree on biopsy, normal values of liver enzymes, transient elastography values consistent with grade 1 fibrosis in both groups, increased waist circumference (WC), a BMI of 30.97 kg/m^2^, and 76% presented with metabolic syndrome (MetS). After the intervention, no differences were observed between the probiotic and placebo groups in terms of MetS, WC, BMI scores, or liver enzyme levels (*p* > 0.05 for all). The elastography values remained consistent with grade 1 fibrosis in both groups. Although CK-18 was reduced in both groups, a larger effect size was noted in the probiotic group (*D* = 1.336). sTLR-4 was also reduced in both groups, with no difference between groups (*p* = 0.885).

**Conclusion:**

Intervention with probiotics in the early stages of NASH demonstrated no significant change in hepatic and clinical parameters.

**Clinical trial registration:**

ClinicalTrials.gov, identifier NCT0346782.

## Introduction

Non-alcoholic fatty liver disease (NAFLD) and its progressive form of non-alcoholic steatohepatitis (NASH) are considered hepatic manifestations of metabolic syndrome (MetS) (1). Recently, NAFLD was renamed metabolic dysfunction-associated steatotic liver disease (MASLD) (2). The disease affects approximately a quarter of the world’s population and is associated with a sedentary lifestyle and a western diet. Thus far, lifestyle changes constitute the first line of treatment, as there is yet no specific pharmacological treatment approved for the disease (1, 2).

It is important to understand the pathogenesis of the disease and the role of the intestinal microbiota in its progression, which may pave the way to discovering new therapies and strategies to control NAFLD. Gut microbiota (GM) has been identified as a potential therapeutic target for patients with NASH (3, 4), as studies have shown changes in the intestinal microbiota of patients with NAFLD and NASH (5–7). A relatively high abundance of genera *Fusobacteria* and a low abundance of genera *Oscillospira* and *Ruminococcus* of the family *Ruminococcaceae* and *Coprococcus* of the family *Lachnospiraceae* were found more in NAFLD patients in parallel with healthy individuals (8). Other bacterial species found in these patients were Proteobacteria, Escherichia, and Enterobacteria (9), and Bacteroides were more common in NASH patients in parallel with healthy individuals (10).

Studies with probiotics have shown benefits in reducing levels of liver enzymes, triglycerides (TGs), low-density lipoprotein cholesterol (LDL-c), steatosis severity, pro-inflammatory cytokines, and even in obesity parameters, such as reduction of visceral and body fat (5, 11, 12). In this context, a recent systematic review followed by a meta-analysis (13), which included 18 RCTs on interventions with probiotic mix (intervention period ranged from 2 to 14 months), demonstrated the effectiveness of these interventions in reducing steatosis (assessed through ultrasound), showing a slight reduction in fibrosis (assessed through elastography), as well as a decrease in levels of aspartate aminotransferase (AST), alanine aminotransferase (ALT), and gamma-glutamyl transferase (GGT). However, the authors state that more studies are necessary due to significant heterogeneity in the probiotic strains used, dosages, and formulations. Another recent systematic review, followed by a meta-analysis (14), evaluated the effect of probiotics on NAFLD parameters and also demonstrated an effect on parameters such as reduction of ALT and AST, serum lipids, glucose, and insulin. In this study, the authors included 21 RCTs, and the intervention time varied from 8 to 56 weeks, with more important effects being observed in studies lasting 12 weeks or longer.

Microbiota dysbiosis is a contributing factor to the disease pathogenesis, related to intestinal barrier dysfunction and increased permeability, exposing the liver to microbial translocation (5). This process activates toll-like receptor 4 (TLR-4), causing an inflammatory response in the liver and initiating the inflammatory cascade (7).

Biopsy is the gold standard for NASH, although it is still considered a risky procedure. Less invasive tests, such as serum cytokeratin 18 (CK-18), have been proposed as promising alternatives to liver biopsy to diagnose NASH and monitor disease progression and response to therapy (15). In addition, scores such as the NAFLD fibrosis score, APRI score, and fat liver index are widely used and have the advantage of not being invasive (16–19). Considering the high prevalence of NASH in the population and the existing gaps in its treatment, this randomized study aimed to evaluate the effect of 24 weeks mix probiotic supplementation [*Lactobacillus acidophilus* (1 × 10^9^ CFU) + *Lactobacillus rhamnosus* (1 × 10^9^ CFU) + *Lactobacillus paracasei* (1 × 10^9^ CFU) + *Bifidobacterium lactis* (1 × 10^9^ CFU)] on liver function markers and clinical parameters in NASH patients.

## Methods

This study is a single-center, randomized, double-blind, placebo-controlled clinical trial that included adult subjects at an outpatient clinic of the Gastroenterology and Nutrition and Dietetic Division of Hospital de Clínicas de Porto Alegre, Brazil. The study included only patients with proven liver biopsy NASH (NAS ≥4), defined as the presence of ≥5% of hepatic steatosis and inflammation with hepatocyte injury, with or without fibrosis, and the absence of causes for secondary hepatic fat accumulation, according to the American Association for the Study of Liver Diseases (AASLD) (1). We investigated the history of alcohol use, hemochromatosis, or Wilson disease, and the history of hepatotoxic drug use. Patients who met the inclusion criteria were enrolled after signing written informed consent. Patients with human immunodeficiency virus, hepatitis B or hepatitis C virus infection, cirrhosis, pregnancy, liver transplantation, supplements and foods with probiotics, immunosuppressants, antibiotic use in the last 6 months, and any other chronic inflammatory diseases were excluded.

This study was conducted according to the guidelines laid down in the Declaration of Helsinki, and all procedures involving human subjects were approved by the ethics and research committee of the *Hospital de Clínicas de Porto Alegre* (Approval Number 16-0438). The protocol was registered at *ClinicalTrials.gov* under the identifier NCT03467282. Written informed consent was obtained from all subjects.

### Intervention

Patients were randomized to an intervention or a placebo group in a numerical sequence on the *randomization.com* website. Patients from the intervention group received probiotic supplementation consisting of a 1 g-sachet containing *Lactobacillus acidophilus* NCFM (1 × 10^9^ CFU) + *Lactobacillus rhamnosus* HN001 (1 × 10^9^ CFU) + *Lactobacillus paracasei* LPC-37 (1 × 10^9^ CFU) + *Bifidobacterium lactis* HN019 (1 × 10^9^ CFU). Patients from the control group received a 1 g sachet with an identical appearance (physical and organoleptic) containing polydextrose/maltodextrin as the placebo. They were instructed to ingest two sachets diluted in water at room temperature daily, before the first meal of the day, for 24 weeks.

During the entire clinical trial period, including the data analysis stage, patients and investigators were blinded to the composition of the sachet content. Only an external researcher had access to this information, and it was only revealed at the end of the study which group each patient belonged to.

All the patients received a spreadsheet to mark the intake of the sachets and write down any symptoms to control their adherence to the treatment. Patients who completed 90% of the recommended treatment were considered in compliance.

In the first visit, patients received general guidance on nutrition, not an individualized plan, and were also instructed to maintain their usual level of physical activity so that this would not be a confounding factor for later interpretation of the results of the intervention. Subsequently, in intermediate visits (days 45, 90, and 135), patients were encouraged to talk about food consumption and daily activities in the last few days in order to identify any changes in the pattern. At these moments, it was again reinforced that the usual standard was maintained.

**Figure 1:**
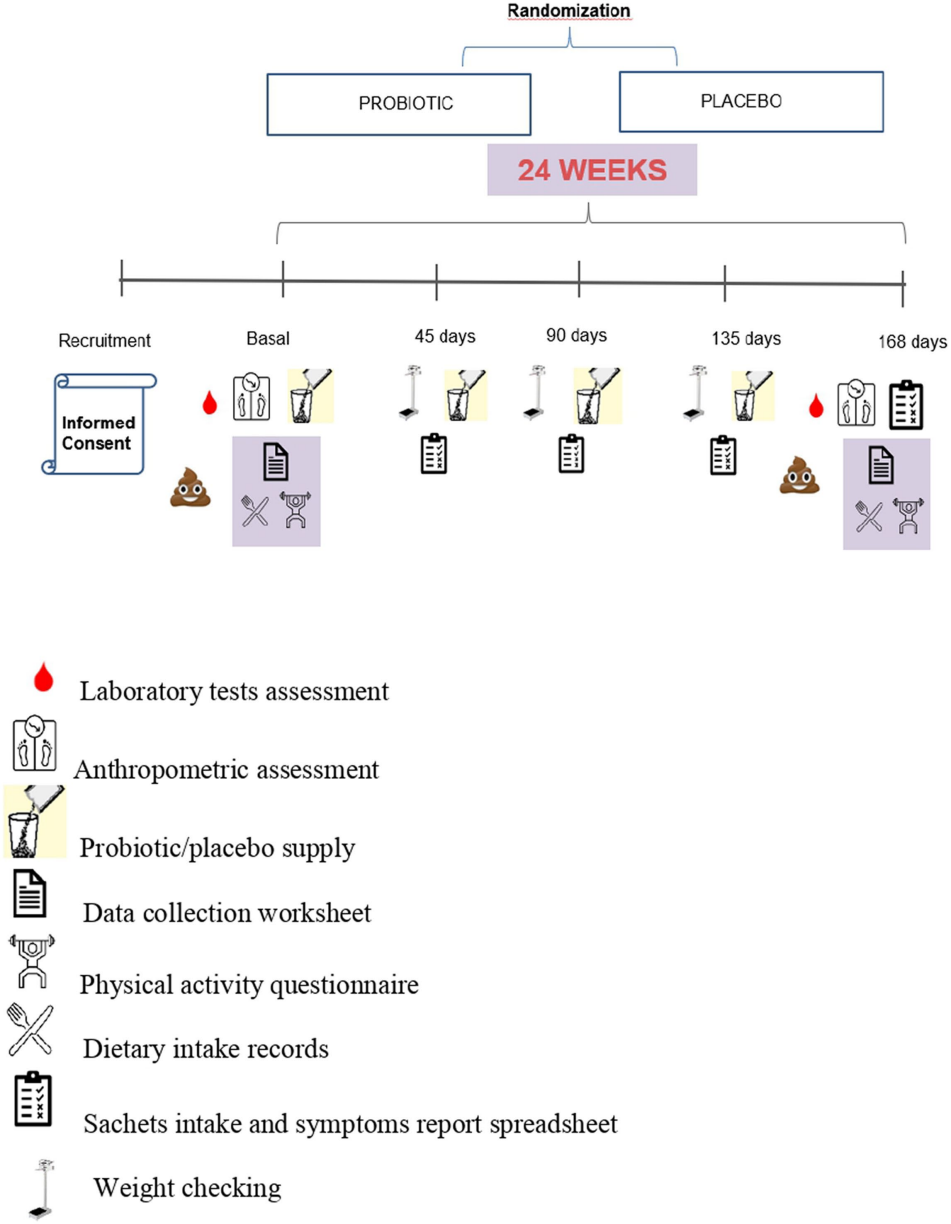
Study’s logistics—detailing participants’ recruitment, randomization, allocation, and methods.

Figure 1 schematically summarizes the study’s logistics, including recruitment, randomization, patients’ visiting frequency, and procedures performed at each moment. Full details of the trial protocol and study design have been previously described (20, 21).

### Clinical evaluation

The data collection protocol includes demographic and clinical data, medications in use, alcohol intake (with significant intake defined as >20 g/day for men and >10 g/day for women) (22), and smoking status. In addition, details of the disease diagnosis (liver biopsy and transient elastography) were recorded.

The MetS was defined as the presence of ≥3 of the criteria presented according to the *International Diabetes Federation, American Heart Association,* and *National Heart Institute* (IDF/AHA/NHLBI) (23). For waist circumference (WC), we used the database of the ELSA-Brazil study (men ≥92 cm, women ≥86 cm) (24).

### Assessment of fibrosis, liver fat, and liver function markers

Before the beginning of the protocol, all patients underwent a liver biopsy to confirm the diagnosis of NASH and determine the degree of fibrosis. We obtained biopsy results for a maximum of 2 years before the start of the intervention. At baseline and the end of the study, participants underwent transient elastography (FibroScan), measurements of liver enzymes, and assessments of serum concentrations of CK-18 and sTLR-4. In addition, the scores were also applied to determine the risk of fibrosis and steatosis.

Transient elastography (FibroScan) was performed in all the patients, and the fibrosis degree was assessed and measured in kilopascals (kPa). The higher the velocity, the greater the stiffness, and the greater the extent of the fibrosis (25, 26). We only considered examinations with ≥10 reliable results, median interquartile range (IQR) values below 30%, and results of transient elastography up to a maximum of 6 months before the intervention. Experienced gastroenterologists performed the imaging examinations (MM and SB).

The immunoenzymatic assay method (ELISA) determined the serum concentration of sTLR-4 (Elabscience, United States) and CK-18 (Elabscience, United States). A spectrophotometer measured the absorbance at a wavelength of 450 nm (Zenyth 200 rt), and the results were expressed in ng/mL and mIU/mL for sTLR-4 and CK-18, respectively. All the processes were performed according to the manufacturer’s instructions, and the analyses were duplicated.

Liver function was assessed through laboratory tests. AST, ALT, GGT, bilirubin, and alkaline phosphatase were measured in the HCPA Laboratory routine on the Cobas Mira Plus equipment through a commercial kit.

To assess liver fibrosis risk, we performed the NAFLD fibrosis score, based on age, BMI, presence of diabetes mellitus (DM) or impaired fasting glucose, AST, ALT, platelet, and albumin levels, and the APRI score, which includes AST and platelet count. The steatosis risk was assessed by the fatty liver index (FLI), using BMI, WC, GGT, and TGs levels. The scores were calculated using MDCalc.com.

### Lipidic, glycemic, and inflammatory profile assessment

The laboratory assessment included measuring lipid profiles [TGs, total cholesterol (TC), HDL cholesterol (HDL), and LDL cholesterol (LDL)] using the colorimetric enzymatic method; levels of other relevant indices such as C-reactive protein (CRP) were measured by nephelometry using a Bayer^®^ nephelometer; insulin levels were measured by electrochemiluminescence (Elecsys 2010 Equipment); glucose was measured by the colorimetric enzymatic method glucose-peroxidase-Biodiagnostic Kit; homeostasis model assessment of insulin resistance (HOMA-IR) was calculated; albumin and creatinine were measured using a commercial kit in the Cobas Mira Plus equipment. Serum was obtained by centrifugation and stored at −80°C. Quantification of the biomarkers was performed using ELISA according to the manufacturer’s instructions.

### Anthropometric assessment

The anthropometric assessment included weight and height measurements for calculating the body mass index (BMI). The WC was measured between the last rib and the iliac crest with an inextensible fiberglass tape measure.

### Dietary intake assessment

Dietary intake was checked by a 3 days diet record, describing the foods eaten on three non-consecutive days (two weekdays and one weekend day). The patients were oriented on filling it correctly, portion sizes, and details of the consumed foods. The NutriBase® software, 2007, calculated the records.

### Physical-activity level

The study applied the International Physical Activity Questionnaire—short form (IPAQ) to evaluate the weekly time spent on physical exercise. The values were expressed as metabolic equivalent of tasks (METs) in minutes per week (27).

### Statistical analysis

The sample size estimation was carried out in WINPEPI 11.20 (Brixton Health, Israel), based on the data from Eslamparast et al. (28) which found a mean reduction in fibrosis score from 9.36 ± 1.9 to 6.38 ± 1.5 in NAFLD patients taking symbiotic supplementation (*p* < 0.001, compared to placebo).

Regarding data treatment, categorical variables were expressed as absolute frequency (*n*) and relative frequency (%), and quantitative variables were expressed as the mean ± SD or median and IQRs (25th to 75th percentile). To check the changes that occurred in each group over the 24 weeks, the paired *t*-test or Wilcoxon test was used. Finally, the comparison between the groups was based on the delta of the difference of each variable analyzed (value at the end of treatment minus the baseline value) using the *t*-test for independent samples or the Mann–Whitney *U*-test.

To verify the magnitude of the effect size (*D*) in the probiotic group and the placebo group, the following reference values were considered: from 0.2 to 0.4 (small effect), from 0.5 to 0.7 (medium effect), and from 0.8 to ≥1.0 (large effect) (29).

The level of statistical significance was taken as a *p*-value of <0.05. The data were analyzed using the Statistical Package for Social Sciences version 20.0 (SPSS Inc., United States).

## Results

**Figure 2:**
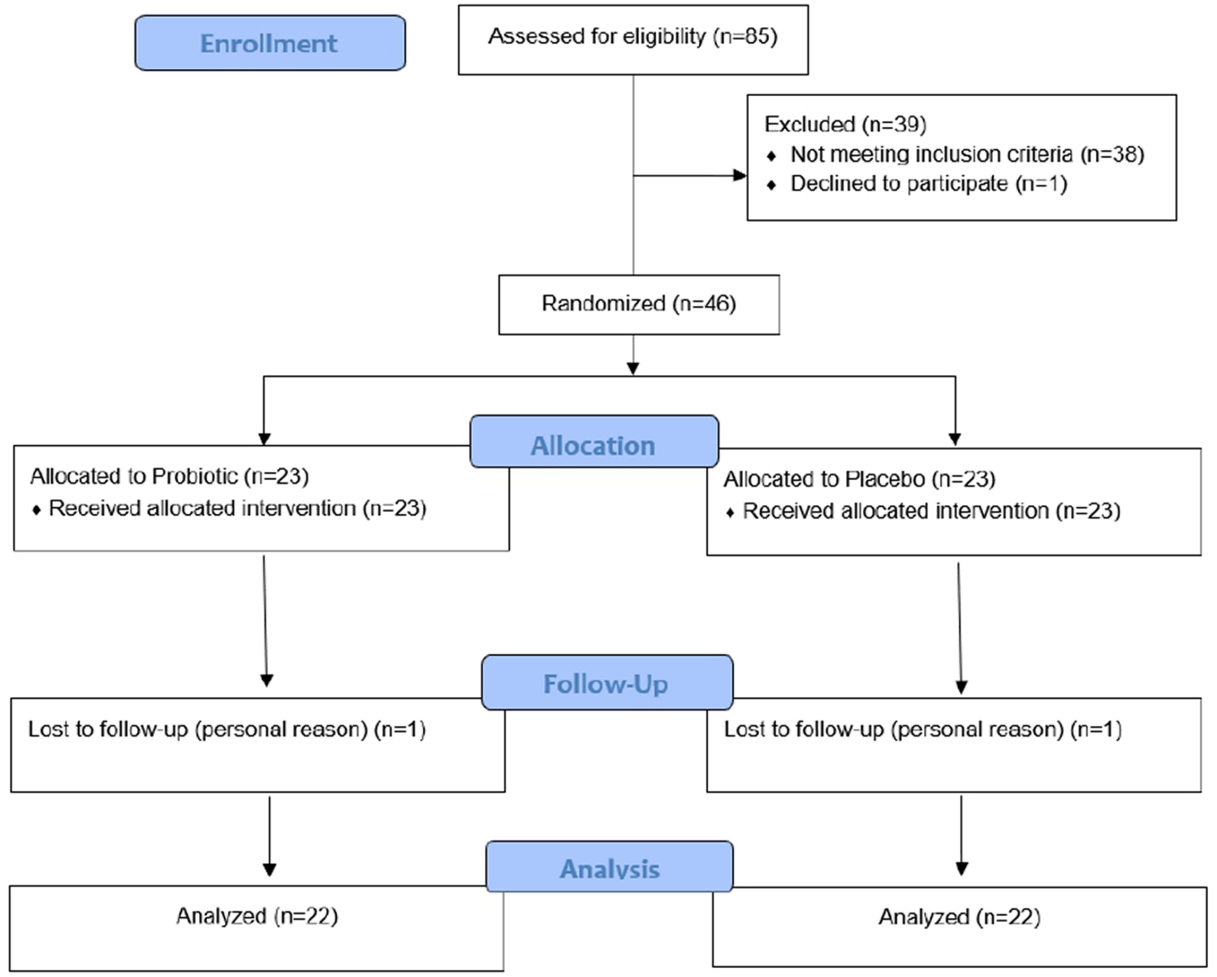
CONSORT flowchart detailing participants’ recruitment, randomization, and allocation.

The flowchart in Figure 2 shows 85 eligible patients who underwent ultrasonography, indicating steatosis and had clinical characteristics compatible with NASH, 39 patients left the trial, 38 patients did not have NASH upon biopsy, and 1 patient withdrew from participating in the research protocol. Therefore, 46 patients with NASH were randomized, and a total of 44 subjects completed the clinical trial and were analyzed.

Table 1 presents the patients’ demographic and clinical baseline data. As for the number of MetS components, at baseline, 16 patients in the probiotic group and 19 patients in the placebo group had three or more components (*p* = 0.428). Over the 24 weeks, this proportion did not change in any group; specifically, 17 (77.3%) vs. 18 (81.9%), *p* = 0.927 (data not shown in table).

**Table 1:** Baseline characteristics of NASH patients.

Variables	All patients (*n* = 46)	Placebo group (*n* = 23)	Probiotic group (*n* = 23)	*p*-value
Female—*n* (%)	27 (58.70%)	12 (52.00%)	15 (65.00%)	0.369
Age (years)	51.35 ± 11.61	51.74 ± 11.94	50.95 ± 11.53	0.983
Waist circumference (cm)	104.70 ± 12.00	104.30 ± 11.00	105.10 ± 13.20	0.807
BMI (kg/m^2^)	30.97 (28.36–33.75)	30.80 (28.36–36.96)	31.14 (28.09–33.75)	0.947
Diabetes mellitus	22.00 (47.80%)	13.00 (56.50%)	9.00 (39.10%)	0.376
High blood pressure	30.00 (65.20%)	14.00 (60.90%)	16.00 (69.60%)	0.758
MetS	35 (76.10%)	19 (82.60%)	16 (69.60%)	0.491
**Number of MetS components—*n* (%)**				
1	3 (6.50%)	2 (8.70%)	1 (4.30%)	0.428
2	8 (17.40%)	2 (8.70%)	6 (26.10%)	
3	14 (30.40%)	8 (34.80%)	6 (26.10%)	
4	10 (21.70%)	4 (17.40%)	6 (26.10%)	
5	11 (23.90%)	7 (30.40%)	4 (17.40%)	
**Degree of fibrosis** ^*^				
F0	12 (26.00%)	8 (34.80%)	4 (17.40%)	0.376
F1	28 (61.00%)	12 (15.52%)	16 (69.60%)	
F2	1 (2.00%)	0 (0%)	1 (4.30%)	
F3	5 (11.00%)	3 (13.00%)	2 (8.70%)	
Glucose (mg/dL)	102.00 (88.00–122.00)	108.00 (97.00–144.00)	94.00 (95.00–115.00)	**0.035**
HbA1c %	5.80 (5.40–6.60)	5.80 (5.30–7.20)	5.70 (5.40–6.40)	0.562
Triglycerides (mg/dL)	145.00 (120.00–230.00)	146.00 (102.00–231.00)	143.00 (121.00–230.00)	0.775
Total cholesterol (mg/dL)	176.00 ± 36.00	173.00 ± 41.00	179.00 ± 32.00	0.604
HDL cholesterol (mg/dL)	45.00 (37.00–52.00)	42.00 (37.00–46.00)	48 (35–53)	0.231
LDL cholesterol (mg/dL)	98.30 ± 31.38	97.13 ± 37.45	99.73 ± 22.88	0.788
Insulin (μUI/mL)	15.1 (12–24.4)	14 (12–23.3)	18.3 (12.1–24.4)	0.512
ALT (U/L)	42 (28–63)	44 (35–63)	36 (22–75)	0.258
AST (U/L)	32 (24–46)	32 (24–44)	29 (22–51)	0.545
GGT (U/L)	46 (28–84)	48 (28–81)	43 (28–105)	0.974
Alkaline phosphatase (U/L)	77 (66–90)	71 (63–85)	80 (68–96)	0.368
Total bilirubin (U/L)	0.50 (0.40–0.70)	0.50 (0.40–0.80)	0.40 (0.30–0.60)	0.219
CRP (g/dL)	2 (1–7)	2 (1–7)	2 (1–5)	0.830
CK-18 (mIU/mL)	841.50 ± 320.07	757.95 ± 343.87	925.04 ± 276.95	0.076
sTLR-4 (ng/mL)	9.25 (5.17–16.48)	11.81 (5.17–20.61)	7.75 (4.46–13.35)	0.199

Categorical variables data were expressed as absolute frequency (*n*) and relative frequency (%). Quantitative variables are expressed as the mean ± SD or median and interquartile ranges (25th to 75th percentile). BMI, body mass index; MetS, metabolic syndrome; HbA1 c%, glycosylated hemoglobin; HDL, high-density lipoprotein; LDL, low-density lipoprotein; ALT, alanine aminotransferase; AST, aspartate aminotransferase; GGT, gamma-glutamyltransferase; CRP, C-reactive protein; CK-18, cytokeratin 18; sTLR-4, soluble toll-like receptor-4; ^*^liver biopsy. Reference values for normality—ALT (♀ < 33 U/L; ♂ < 41 U/L); AST (♀ < 32 U/L; ♂ < 40 U/L); GGT (♀ < 40 U/L; ♂ < 60 U/L); alkaline phosphatase (♀: 35–104 U/L; ♂: 40–129 U/L); total bilirubin <1.2 mg/dL.

Table 2 shows the clinical characteristics and biochemical parameters before and after 24 weeks. There were no changes in the NAFLD fibrosis score, APRI score, or FLI after the intervention, whereas the biomarkers, CK-18, and sTLR-4 were reduced in both. However, there was no statistical difference between the groups. CK-18 showed a reduction in the probiotic group, and this result was subjected to effect size analysis, finding a large effect (*D* = 1.336) when compared to the placebo group (*D* = 0.536). Regarding the sTLR-4 analysis, the values showed a smaller difference (*D* = 0.476 in the probiotic group and *D* = 0.510 in the placebo group).

**Table 2:** Clinical characteristics of NASH patients before and after intervention.

Variables	Baseline	After 24 weeks	*p*-value	Mean change deltas	*p*-value^c^
**Transient elastography (kPa)** ^*^					
Probiotic group (*n* = 20)	7.80 (6.20–11.20)	7.40 (5.60–11.10)	0.575^a^	−0.40 [(−1.20) to 1.30]	0.577
Placebo group (*n* = 17)	7.70 (5.80–9.50)	6.90 (6.40–10.50)	0.244^b^	−0.50 [(−1.90) to 0.70]	
**NAFLD fibrosis score**					
Probiotic group (*n* = 21)	−1.81 ± 1.52	−1.57 ± 1.57	0.053^a^	0.23 ± 0.52	0.612
Placebo group (*n* = 22)	−1.30 ± 1.51	−1.17 ± 1.32	0.411^b^	0.13 ± 0.75	
**APRI score**					
Probiotic group (*n* = 22)	0.39 (0.28–0.74)	0.44 (0.24–0.56)	0.410^a^	0.02 [(−0.10) to 0.08]	0.565
Placebo group (*n* = 22)	0.48 (0.37–0.62)	0.46 (0.30–0.66)	0.386^b^	−0.02 [(−0.08) to 0.13]	
**Fat liver index**					
Probiotic group (*n* = 21)	85.00 (65.00–90.00)	83.00 (52.00–93.00)	0.600^a^	0.00 [(−11) to 8.00]	0.534
Placebo group (*n* = 19)	89.00 (75.00–95.50)	88.00 (73.50–97.00)	0.655^b^	0.00 [(−5.00) to 1.00]	
**CK-18 (mIU/mL)**					
Probiotic group (*n* = 22)	913.75 ± 277.10	640.83 ± 246.85	**<0.001** ^a^	−272.91 ± 204.29	0.109
Placebo group (*n* = 22)	769.50 ± 323.48	610.10 ± 259.10	**0.020** ^b^	−158.53 ± 295.48	0.143
**sTLR-4 (ng/mL)**					
Probiotic group (*n* = 22)	11.02 ± 7.01	8.99 ± 5.36	0.053^a^	−2.03 ± 4.27	0.885
Placebo group (*n* = 22)	13.28 ± 7.84	11.26 ± 6.94	**0.026** ^b^	−2.02 ± 3.10	0.993
**ALT (U/L)**					
Probiotic group (*n* = 22)	39.00 (23.00–75.00)	32.50 (21–71)	0.831^a^	−2 [(−7) to 4]	0.549
Placebo group (*n* = 22)	45.00 (36.00–63.00)	38.50 (32–48)	0.163^b^	−4 [(−16) to 5]	
**AST (U/L)**					
Probiotic group (*n* = 22)	32.00 (22.00–51.00)	29.00 (22.00–52.00)	0.864^a^	0.50 [(−9) to 3]	0.473
Placebo group (*n* = 22)	32.00 (25.00–44.00)	28.00 (23.00–37.00)	0.114^b^	−3.50 [(−10) to 2]	
**GGT (U/L)**					
Probiotic group (*n* = 22)	40.00 (28.00–94.00)	43.50 (24.00–87.00)	0.776^a^	−1 [(−4) to 3]	0.060
Placebo group (*n* = 20)	50.00 (34.50–82.50)	44.00 (30.50–71.50)	0.378^b^	−5 [(−9) to 0.50]	
**Alkaline phosphatase (U/L)**					
Probiotic group (*n* = 22)	79.00 (68.00–92.00)	73.00 (57.00–91.00)	0.325^a^	−4 [(−8) to 2]	0.481
Placebo group (*n* = 22)	71.00 (63.00–85.00)	72.00 (59.00–77.00)	0.172^b^	−4 [(−10) to 1]	
**Total bilirubin (mg/dL)**					
Probiotic group (*n* = 22)	0.45 (0.30–0.60)	0.45 (0.30–0.60)	0.684^a^	0 [(−0.10) to 0.10]	0.605
Placebo group (*n* = 21)	0.50 (0.40–0.70)	0.50 (0.40–0.70)	0.256^b^	0 [(−0.10) to 0.10]	
**Direct bilirubin (mg/dL)**					
Probiotic group (*n* = 22)	0.20 (0.10–0.30)	0.20 (0.20–0.20)	0.414^a^	0 (0–0)	0.818
Placebo group (*n* = 21)	0.20 (0.20–0.30)	0.20 (0.20–0.30)	0.180^b^	0 (0–0)	
**Indirect bilirubin (mg/dL)**					
Probiotic group (*n* = 22)	0.30 (0.20–0.30)	0.30 (0.20–0.40)	0.666^a^	0 [(−0.10–0.10)]	0.580
Placebo group (*n* = 21)	0.30 (0.20–0.50)	0.30 (0.20–0.40)	0.465^b^	0 [(−0.10–0.10)]	
**Insulin (μUI/mL)**					
Probiotic group (*n* = 21)	17.80 (12.10–22.10)	14.60 (13.20–20.00)	0.850^a^	0.50 [(−3.40) to 2.40]	0.715
Placebo group (*n* = 20)	14.20 (12.40–25.90)	13.05 (10.05–23.15)	0.836^b^	−0.25 [(−3.90) to 2.30]	
**HOMA-IR**					
Probiotic group (*n* = 22)	74.34 (48.78–104.86)	65.83 (48.32–114.92)	0.332^a^	2.91 [(−10.53) to 18.53]	0.339
Placebo group (*n* = 20)	77.49 (58.98–105.40)	68.86 (53.64–117.99)	0.877^b^	−0.04 [(−34.71) to 14.96]	
**Glucose (mg/dL)**					
Probiotic group (*n* = 22)	95 (85–115)	98.50 (86–128)	0.850^a^	−1.50 [(−10) to 12]	0.543
Placebo group (*n* = 21)	109 (100–144)	115 (99–134)	0.532^b^	−4 [(−13) to 10]	
**Triglycerides (mg/dL)**					
Probiotic group (*n* = 22)	142.00 (121.00–207.00)	172.50 (114.00–201.00)	0.287^a^	4.50 [(−30) to 41]	0.366
Placebo group (*n* = 22)	144.00 (102.00–230.00)	135.00 (112.00–184.00)	0.587^b^	−15 [(−44) to 34]	
**Total cholesterol (mg/dL)**					
Probiotic group (*n* = 22)	179.00 ± 32.00	184.24 ± 42.49	0.194^a^	4.57 ± 21.99	0.392
Placebo group (*n* = 22)	173.00 ± 42.00	172.23 ± 44.17	0.375^b^	−1.50 ± 23.99	
**HDL cholesterol (mg/dL)**					
Probiotic group (*n* = 22)	48.00 (35.00–53.00)	45.50 (41.00–56.00)	0.897^a^	1 [(−3) to 3]	1.000
Placebo group (*n* = 22)	42.00 (37.00–46.00)	43.50 (38.00–47.00)	0.815^b^	1 [(−5) to 5]	
**LDL cholesterol (mg/dL)**					
Probiotic group (*n* = 17)	101.19 ± 22.71	105.28 ± 28.11	0.733^a^	−1.36 [(−8.24) to 11.88]	0.794
Placebo group (*n* = 21)	98.42 ± 37.86	97.44 ± 36.74	0.475^b^	0.34 [(−8.70) to 11]	
**Creatinine (mg/dL)**					
Probiotic group (*n* = 21)	0.83 ± 0.20	0.83 ± 0.18	0.384^a^	0.02 [(−0.02) to 0.04]	0.782
Placebo group (*n* = 21)	0.81 ± 0.16	0.82 ± 0.16	0.520^b^	0.01 [(−0.03) to 0.05]	
**Albumin (g/dL)**					
Probiotic group (*n* = 19)	4.70 ± 0.40	4.60 ± 0.26	0.228^a^	−0.10 ± 0.26	0.812
Placebo group (*n* = 20)	4.70 ± 0.30	4.64 ± 0.29	0.064^b^	−0.05 ± 0.24	
**CRP (g/dL)**					
Probiotic group (*n* = 19)	2.00 (1.00–4.00)	3.10 (1.20–4.30)	0.856^a^	−0.10 [(−0.60) to 1.10]	0.339
Placebo group (*n* = 19)	3.00 (2.00–8.00)	4.10 (1.70–7.20)	0.593^b^	0.40 [(−0.70) to 3.95]	
**Platelets (μL)**					
Probiotic group (*n* = 21)	240.00 ± 58.00	237.29 ± 62.06	0.144^a^	−2.67 ± 19.63	0.258
Placebo group (*n* = 21)	226.00 ± 55.00	214.10 ± 53.45	0.364^b^	−11.90 ± 31.24	

Variables are expressed as the mean ± SD or median and interquartile ranges (25th to 75th percentile). The mean change deltas may not total the subtraction because of the normality of the variable. ^*^kPa, klilopascals—FibroScan; CK-18, cytokeratin 18; sTLR-4, soluble toll-like receptor-4; ALT, alanine aminotransferase; AST, aspartate aminotransferase; GGT, gamma-glutamyltransferase; HOMA-IR, homeostasis model assessment of insulin resistance; HDL, high-density lipoprotein; LDL, low-density lipoprotein; CRP, C-reactive protein. ^a^*p*-value referring to the changes that occurred within the probiotic group over the 24 weeks (paired *t*-test or Wilcoxon test). ^b^*p*-value referring to the changes that occurred within the placebo group over the 24 weeks (paired *t*-test or Wilcoxon test). ^c^*p*-value mean change deltas between groups (*t*-test for independent samples or Mann–Whitney *U*-test).

Table 3 presents the anthropometric characteristics before and after the intervention. No differences between the groups were observed after treatment.

**Table 3:** Anthropometric characteristics of NASH patients before and after intervention.

Variables	Baseline	After 24 weeks	*p*-value	Mean change deltas	*p*-value^c^
**Weight (kg)**					
Probiotic group	83.45 ± 16.50	83.64 ± 15.55	0.726^a^	0.50 (0–1)	0.274
Placebo group	87.41 ± 17.59	87.23 ± 18.16	0.702^b^	0 [(−2) to 1]	
**BMI (kg/m** ^**2**^ **)**					
Probiotic group	30.92 (28.09–33.24)	30.70 (28.01–33.75)	0.524^a^	0.18 (0.00–0.43)	0.219
Placebo group	31.16 (29.05–36.92)	30.81 (28.70–35.70)	0.714^b^	0.00 [(−0.70) to 0.43]	
**Waist circumference (cm)**					
Probiotic group	103.6 ± 11.20	103.52 ± 10.12	0.913^a^	−0.08 ± 3.28	0.875
Placebo group	104.7 ± 11.10	104.76 ± 11.06	0.905^b^	0.05 ± 2.12	

Variables are expressed as the mean ± SD or median and interquartile ranges (25th to 75th percentile). The mean change deltas may not total the subtraction because of the normality of the variable. BMI, body mass index. ^a^*p*-value referring to the changes that occurred within the probiotic group over the 24 weeks (paired *t*-test or Wilcoxon test). ^b^*p*-value referring to the changes that occurred within the placebo group over the 24 weeks (paired *t*-test or Wilcoxon test). ^c^*p*-value mean change deltas between groups (*t*-test for independent samples or Mann–Whitney *U*-test).

Table 4 shows the patients’ dietary intake characteristics before and after the intervention. There was a significant reduction in energy, carbohydrates, proteins, and lipid intake in the probiotic group and a significant reduction just in cholesterol intake in the placebo group, but when one group was compared to the other, these differences were not significant.

**Table 4:** Diet of NASH patients before and after intervention.

Variables	Baseline	After 24 weeks	*p*-value	Mean change	*p*-value^c^
**Energy intake (kcal/day)**					
Probiotic group (*n* = 17)	2286.71 (2054.96–2516.22)	1758.38 (1366.91–2156.79)	**0.013** ^a^	−351 ± 494.03	0.744
Placebo group (*n* = 16)	2286.67 (1708.74–3071.52)	2139.47 (1920.46–2442.13)	0.278^b^	−289.00 ± 716.56	
**Carbohydrates (g/day)**					
Probiotic group (*n* = 17)	248.50 (221.50–30.9.53)	195.00 (158.38–233.90)	**0.039** ^a^	−50.89 ± 94.45	0.892
Placebo group (*n* = 16)	289.40 (208.59–381.27)	255.79 (216.86–307.35)	0.148^b^	−46.28 ± 89.33	
**Protein (g/kg/day)**					
Probiotic group (*n* = 17)	1.40 ± 0.47	1.06 ± 0.30	**0.014** ^a^	−0.33 ± 0.50	0.374
Placebo group (*n* = 16)	1.26 ± 0.44	1.12 ± 0.34	0.258^b^	−0.15 ± 0.50	
**Fat (g/day)**					
Probiotic group (*n* = 17)	86.88 (62.74–92.96)	85.81 (74.70–131.46)	**0.049** ^a^	−13.08[(−53.60) to (0.08)]	0.581
Placebo group (*n* = 16)	100.27 (61.98–120.67)	82.01 (74.12–102.29)	0.278^b^	3.11 [(−53.58) to 21.92]	
**Cholesterol (mg/day)**					
Probiotic group (*n* = 17)	344.14 ± 157.71	284.99 ± 141.94	0.263^a^	−59.16 ± 210.26	0.857
Placebo group (*n* = 16)	354 ± 163.23	285 ± 117.71	**0.018** ^b^	−69.59 ± 105.38	
**Total fibers (g/day)**					
Probiotic group (*n* = 17)	17.76 (14.13–25.62)	15.30 (14.22–17.46)	0.124^a^	−4.73 ± 11.19	0.265
Placebo group (*n* = 16)	17.58 (15.15–30.20)	19.38 (16.56–26.53)	0.756^b^	−0.12 ± 12.12	

Variables are expressed as the mean ± SD or median and interquartile ranges (25th to 75th percentile). The mean change deltas may not total the subtraction because of the normality of the variable. ^a^*p*-value referring to the changes that occurred within the probiotic group over the 24 weeks (paired *t*-test or Wilcoxon test). ^b^*p*-value referring to the changes that occurred within the placebo group over the 24 weeks (paired *t*-test or Wilcoxon test). ^c^*p*-value mean change deltas between groups (*t*-test for independent samples or Mann–Whitney *U*-test).

The physical activity was expressed as METs in minutes per week, showing no difference between the groups throughout the study (*p* = 0.752). In the probiotic group, the baseline median was 834 (300–2,118) min, and after the intervention, 773 (596–2,718) min (*p =* 0.217). In the placebo group, the baseline median was 1,060 (299–1,794) min, and after the intervention,1,020 (678–2,810) min (*p* = 0.181).

## Compliance and adverse effects

One patient in the probiotic group was considered non-compliant with the protocol; however, they were included in the intention-to-treat analysis. There were no reports of adverse events associated with the probiotic supplementation during the entire trial.

## Discussion

NASH is currently known as the leading cause of liver transplantation. Its prevalence has increased along with the worldwide increase in obesity (3, 30). Measures are necessary both to prevent the onset of NASH as well as to treat associated complications and prevent its progression to more severe forms (31, 32). The purpose of the present study was to evaluate the effect of 24 weeks probiotic supplementation in NASH patients on liver function markers, nutritional status, and clinical parameters. The 24 weeks intervention with probiotics demonstrated that they do not promote a significant change in liver and clinical parameters.

Despite being young (average age 51 years), NASH patients’ baseline demographic and clinical data showed increased WC, obesity, hypertension, and DM. These typical characteristics were present in almost 80% of the patients. There was no difference between the groups, showing the homogeneity of the sample to start the treatment, except for the lower glucose level in the group of probiotics, in which fewer patients were diagnosed with DM.

The patients’ routine diet and usual physical activities were assessed before and after treatment to ensure none of these factors interfered with the results. Although the dietary intervention was not our target, especially because we emphasized that patients should not change their consumption pattern over the 24 weeks, the fact that they returned on days 45, 90, and 135 for follow-up probably caused “greater attention.” The research nutritionists were the ones providing the care, and this may have made them pay more attention to what they were eating. It is an unintentional effect over which we had no control since patients ate their meals in their own homes. Although all patients only received general dietary guidelines at the beginning of the study, they probably began to be more careful with their food choices, which may have affected the results. From a methodological point of view, with appropriate statistical treatment, it was observed that there was a significant reduction in the intake of calories, carbohydrates, proteins, and lipids in the probiotic group, while in the placebo group, only cholesterol intake showed a significant reduction. However, when the groups were compared to each other, these differences were not significant, that is, although there was a reduction in food intake, it was similar in both groups. This unintentional improvement in diet, which occurred in both groups, may have been the reason why it was not possible to observe differences in other parameters evaluated. Concerning physical activity, no difference between the groups was observed.

The RCTs with probiotics are quite heterogeneous, both in terms of intervention time, type of strains, and the amount administered (5, 13, 14, 33–35). These differences between studies do not allow a generalized indication of probiotics as a treatment adjunct (36). There are a lot of different strains, and the effects resulting from their administration can also be quite different (11). Probiotics are expected to reconstitute a healthy microbiome, but the number of bacteria existing in the intestine is far greater than that offered by probiotics in the form of supplements (36). It really makes sense to consider that the human intestine is inhabited by approximately 100 trillion bacteria, viruses, and fungi, forming a truly diverse ecosystem (5, 37). Another important key point concerns the use of combinations of bacteria species at the expense of the use of a single strain. In a systematic review with 22 RCTs using probiotics in NAFLD patients (34), 77.8% of interventions were carried out with a mix of probiotics, and only 4 studies were carried out with a single strain. The justification for a preferred prescription of a mixture of probiotics is that different strains have achieved better outcomes in RCTs and can act on different targets, achieving better results in reducing steatosis, fibrosis, AST, ALT, serum lipids, glucose, and insulin (5, 11, 13, 14, 34, 35, 38).

Despite the use of an accessible mixture of probiotic strains and a high supplemented amount, the expected effects were either very modest or not achieved. The biochemical levels of patients with NASH may be altered more according to the severity of the disease (39). However, from a clinical point of view, considering baseline examinations, the patients included in the study were not poorly managed. Additionally, in relation to the degree of fibrosis and hepatic impairment, most patients had low fibrosis or levels close to normal (stage 1). These reasons may explain the absence of significant findings, as the treatment might have had limited effectiveness in reducing these parameters, including liver enzymes.

Despite being the gold standard for NASH diagnosis, liver biopsy is an invasive method that causes discomfort and may have some risks (1). Thus, non-invasive tests such as scores and CK18 for screening and risk stratification of advanced liver fibrosis have gained increasing visibility (15–18, 40). CK-18 is the major intermediate filament protein comprising the cytoskeletal structure of hepatocytes. It turns out that during hepatocyte apoptosis, the effector caspase cleaves fragments of CK-18 in the bloodstream (41), thus making it possible to evaluate its serum concentration. A recent study (42) evaluated circulating levels of CK-18 in more than 1,000 patients from different centers who had a liver biopsy (153 with NAFL and 855 with NASH). There was an interaction between CK-18 levels and serum AST and BMI. Furthermore, CK-18 showed a positive association with histological NAS, but with unsatisfactory sensitivity and positive predictive value (55 and 59%, respectively), which led the authors to conclude that the isolated measurement of CK-18 would have limited value for the non-invasive diagnosis of NASH. In the present RCT, CK-18 concentration was evaluated and reduced after intervention in both groups. Although the probiotic group had a greater reduction in CK-18 values and a larger effect size, there was no difference between the groups. We believe that intensive monitoring during the protocol meant that the patients were more careful with their diet over the 24 weeks, as both showed a reduction in calorie intake and macronutrients even without statistical differences between them.

We identified that there was a decrease in sTLR-4 after the intervention, but there was no significant difference between the groups. TLR-4 is a key immunological pathway activated by bacterial lipopolysaccharides that produce pro-inflammatory cytokines, inducing metabolic disorders and promoting greater liver damage in patients with NAFLD (43, 44). A recent review addressed the role of TLR-4 in the development of NAFLD through the activation of the immune response of endotoxin-producing strains (*Enterobacter cloacae* B29, *Escherichia coli* PY102, and *Klebsiella pneumoniae* A7) (45).

These receptors can recognize a variety of stimuli and thus initiate an immunological response through the formation of a protein complex. It is important to point out that all parallel TLR pathways compete and thus restrict each other’s activation due to overlapping binding sites (46). A limitation of our study was that the evaluation was restricted to the soluble form of TLR-4. This soluble form might even be considered an anti-inflammatory marker since these soluble receptors neutralize ligands and are negative regulators of inflammation (46). Ideally, it would be necessary to identify the multiprotein complex to confirm that an inflammatory response is occurring.

After 24 weeks of the intervention, it was impossible to perceive a decrease in fibrosis or liver fat through the assessed scores. Following our results, symbiotic supplementation for 12 months also had no impact on the NAFLD fibrosis score (47). However, a 2 years lifestyle intervention resulted in a reduction in the NAFLD fibrosis score, weight, WC, and liver enzymes (48).

Similar to our study, but with a much smaller sample size (49), a study using a probiotic mix twice a day for 24 weeks demonstrated a reduction in the intrahepatic fat content assessed by magnetic resonance imaging. However, the included patients had a degree of fibrosis equal to 2 or 3, had higher baseline values of intrahepatic fat, and were instructed to make lifestyle changes (diet and exercise). However, the glycemic and lipid profiles did not change, as in our study.

As for liver enzymes, unlike other authors, we could not demonstrate any significant reduction after treatment. In a brief report (50), 15 NASH patients with altered hepatic enzymes received *Acidophilus* capsules (2 billion viable organisms) 3 times daily for 1 month. They demonstrated a significant reduction in ALT (*p* < 0,001) and AST (*p* = 0.03) levels when comparing the intervention group and control group; however, there was no alteration in the imaging examinations. Other authors reported a reduction in liver enzymes after the supplementation of probiotics (51–53), but it is worth noting that the baseline values of all these patients were high.

In general, changes in anthropometric measures are not expected with probiotics, and Wong et al. (49) and Behrouz et al. (53) did not find changes in these parameters. However, Alisi et al. (54) demonstrated a reduction in BMI after the supplementation of a high concentration of probiotic strains for 16 weeks and Famouri et al. (55) in WC and the weight of obese children after supplementing a mixture of probiotics for 12 weeks. It is important to emphasize that in these two interventions, in addition to probiotics, the participants (children and adolescents) were prescribed a low-calorie diet or were encouraged to increase their daily activity, as well as improve their eating habits by increasing their intake of fruits and vegetables and reducing their consumption of fast food, high-fat meals, and sweet snacks.

As we did not find a change in the number of Mets components after supplementation, a more extended intervention would probably be necessary to achieve these effects. In this regard, Duseja et al. (56) also did not succeed in this regard after a similar intervention.

This study has some solid points, including being a single-center, RCT, double-blind, and adhering to methodological guidelines. There was a minor loss of patients during the follow-up period. All patients underwent liver biopsy for the NASH diagnosis before starting the protocol and were followed up every 45 days for reassessment and compliance monitoring. Nevertheless, the study presents a limited fibrosis assessment. No liver biopsy was performed at the end of the study, and not all the patients were suitable candidates for *FibroScan* due to BMI or central obesity.

## Conclusion

In conclusion, the 24 weeks intervention with probiotics demonstrated that it does not promote a significant change in liver and clinical parameters for patients in the early stages of NASH. However, its use for disease prevention or in more advanced stages needs to be further evaluated.

## Data availability statement

The raw data supporting the conclusions of this article will be made available by the authors, without undue reservation.

## Ethics statement

The studies involving humans were approved by Comitê de Ética em Pesquisa—Hospital de Clínicas de Porto Alegre. The studies were conducted in accordance with the local legislation and institutional requirements. The participants provided their written informed consent to participate in this study.

## Author contributions

AS-S: Conceptualization, Data curation, Formal analysis, Funding acquisition, Investigation, Methodology, Resources, Writing – original draft. HM: Data curation, Formal analysis, Investigation, Methodology, Resources, Writing – original draft. SB: Investigation, Methodology, Resources, Writing – original draft. BM: Formal analysis, Investigation, Methodology, Writing – original draft. LL: Formal analysis, Investigation, Methodology, Resources, Writing – original draft. MM: Investigation, Methodology, Writing – original draft. CC: Investigation, Methodology, Writing – original draft. CU-C: Conceptualization, Funding acquisition, Methodology, Resources, Writing – original draft. TS: Conceptualization, Funding acquisition, Methodology, Project administration, Resources, Writing – original draft. MÁ-d-S: Investigation, Methodology, Resources, Conceptualization, Writing – original draft. VD: Conceptualization, Data curation, Formal analysis, Funding acquisition, Investigation, Methodology, Project administration, Resources, Supervision, Writing – review & editing.

## Glossary

ALT: Alanine aminotransferase
AST: Aspartate aminotransferase
APRI: Aspartate aminotransferase to platelet ratio index
ASCVD: Atherosclerotic cardiovascular disease
BMI: Body mass index
BMR: Basal metabolism rate
CAP: Controlled attenuation parameter
CFU: Colony-forming units
CK-18: Cytokeratin 18
CRP: C-reactive protein
DM: Diabetes mellitus
GGT: Gamma-glutamyltransferase
GM: Gut microbiota
HbA1c: Glycosylated hemoglobin
HCPA: Hospital de Clinicas de Porto Alegre
HDL: High-density lipoprotein
IPAQ: International Physical Activity Questionnaire
LDL: Low-density lipoprotein
LPS: Bacterial lipopolysaccharide
MASLD: Metabolic dysfunction-associated steatotic liver disease
MetS: Metabolic syndrome
NAFLD: Non-alcoholic fatty liver disease
NASH: Non-alcoholic steatohepatitis
RCT: Randomized clinical trial
TC: Total cholesterol
TG: Triglycerides
TLR: Toll-like receptor
sTLR-4: Soluble toll-like receptor-4
